# Long-Term Continuous Extraction of Medium-Chain Carboxylates by Pertraction With Submerged Hollow-Fiber Membranes

**DOI:** 10.3389/fbioe.2021.726946

**Published:** 2021-08-13

**Authors:** Jiajie Xu, Bin Bian, Largus T. Angenent, Pascal E. Saikaly

**Affiliations:** ^1^ Biological and Environmental Science and Engineering (BESE) Division, Water Desalination and Reuse Center (WDRC), King Abdullah University of Science and Technology, Thuwal, Saudi Arabia; ^2^ Environmental Biotechnology Group, Center for Applied Geosciences, University of Tübingen, Tübingen, Germany

**Keywords:** chain elongation, anaerobic fermentation, carboxylate extraction, submerged membrane, liquid-liquid extraction

## Abstract

Medium-chain carboxylic acids (MCCAs), which can be generated from organic waste and agro-industrial side streams through microbial chain elongation, are valuable chemicals with numerous industrial applications. Membrane-based liquid-liquid extraction (pertraction) as a downstream separation process to extract MCCAs has been applied successfully. Here, a novel pertraction system with submerged hollow-fiber membranes in the fermentation bioreactor was applied to increase the MCCA extraction rate and reduce the footprint. The highest average surface-corrected MCCA extraction rate of 655.2 ± 86.4 mmol C m^−2^ d^−1^ was obtained, which was higher than any other previous reports, albeit the relatively small surface area removed only 11.6% of the introduced carbon *via* pertraction. This submerged extraction system was able to continuously extract MCCAs with a high extraction rate for more than 8 months. The average extraction rate of MCCA by internal membrane was 3.0- to 4.7-fold higher than the external pertraction (traditional pertraction) in the same bioreactor. A broth upflow velocity of 7.6 m h^−1^ was more efficient to extract MCCAs when compared to periodic biogas recirculation operation as a means to prevent membrane fouling. An even higher broth upflow velocity of 40.5 m h^−1^ resulted in a significant increase in methane production, losing more than 30% of carbon conversion to methane due to a loss of H_2_, and a subsequent drop in the H_2_ partial pressure. This resulted in the shift from a microbial community with chain elongators as the key functional group to methanogens, because the drop in H_2_ partial pressure led to thermodynamic conditions that oxidizes ethanol and carboxylic acids to acetate and H_2_ with methanogens as the syntrophic partner. Thus, operators of chain elongating systems should monitor the H_2_ partial pressure when changes in operating conditions are made.

## Introduction

Carbon recovery from organic waste or wastewater is attractive to achieve a circular economy as part of a sustainable development, because it not only reduces the cost of waste treatment but also increases the recoverable chemical energy (Lu et al., 2018; Hao et al., 2019). One of the biotechnology production platforms that is of interest for renewable chemical production is microbial chain elongation. Chain elongation harnesses the potential of certain microbes in anaerobic fermentation biotechnology to generate medium-chain carboxylic acids (MCCAs, C6-C12) through a microbial fermentation pathway with an electron acceptor, such as short-chain carboxylic acids (SCCAs, C2-C5), and an electron donor (e.g., ethanol or lactic acid), which can be obtained through the hydrolysis of organic biomass (Angenent et al., 2016; Xu et al., 2018; Daly et al., 2020) or added to the waste. The pathway of reverse β-oxidation is considered a thermodynamically favorable microbial fermentation pathway to produce MCCAs (Dellomonaco et al., 2011; González-Cabaleiro et al., 2013). MCCAs are valuable molecules and are utilized for various industrial and agricultural applications such as sustainable antimicrobials (Kim and Rhee, 2013), precursors for liquid biofuel production (Urban et al., 2017), oleochemical production (Zhu et al., 2020), ionic liquid for protein separation (Lee et al., 2017), and livestock feed additives for growth (Mills et al., 2010). However, it is challenging to reach a high concentration of MCCAs in the microbial fermentation system due to the cellular toxicity of MCCAs (Zhu et al., 2020). The uncharged carboxylic acids disrupt the cell membrane and these acids with longer carbon chains up to eight carbon chains are more toxic due to the increased hydrophobicity of the carbon chain (Butkus et al., 2011; Harroff et al., 2017). Currently, the in-line pertraction system for MCCA extraction is considered one of the best options for reducing cell membrane toxicity and end-product feedback inhibition, thus, enabling high MCCAs production rates (Michel-Savin et al., 1990; Roe et al., 2002; Červeňanský et al., 2019; Lambrecht et al., 2019).

Several technologies have been applied for the in-line removal of MCCAs directly from the fermentation broth, including electrodialysis (Wang et al., 2013; López-Garzón and Straathof, 2014), permeation (Zhu et al., 2021), electrolysis (Carvajal-Arroyo et al., 2020), electrodialysis/phase separation (Xu et al., 2021), and membrane-based liquid-liquid extraction (i.e., pertraction) (Ge et al., 2015; Xu et al., 2015; Saboe et al., 2018; Gehring et al., 2020). Pertraction for in-line extraction of MCCAs has been well studied due to: 1) its low energy cost (mainly requiring electric power to pump the fermentation broth, hydrophobic solvent, and pertraction solution); and 2) selective extraction of the longest possible carbon chain of carboxylate (Angenent et al., 2018). The driving force for MCCA pertraction is a pH gradient (∼5.0–9.0) to specifically extract uncharged carboxylic acids by diffusion through a forward and a backward membrane (Angenent et al., 2018). In accordance with a previous pertraction study (Kucek et al., 2016b), an increase in the recycle flow rates of fermentation broth (0–225 m d^−1^) through the forward membrane module led to an increase in the MCCA mass transfer rate. However, increasing the recycle flow rates of the hydrophobic solvent or the alkaline extraction solution did not affect the overall mass transfer rates, indicating that mass transfer limitations were at the interface of the fermentation broth and the hydrophobic membrane contactor.

The submerged membrane bioreactor (MBR) concept has been widely applied to treat wastewater (Ueda et al., 1997; Wu et al., 2020). Submerged hollow-fiber MBR systems provide self-support and the high surface-area-to-volume ratio and compactness that is needed for large-scale applications (Yoon et al., 2004; Yang et al., 2013; Katuri et al., 2016; Qin et al., 2020). However, membrane fouling still is one of the most important challenges for submerged MBR applications (Lu et al., 2016; Matar et al., 2017; Meng et al., 2020). To delay membrane fouling, operators use scouring of the outer membrane surface through gas sparging and inducing surface shear force through liquid upflow velocity (Fulton et al., 2011; Ozgun et al., 2013; Vermaas et al., 2014). Besides wastewater treatment, submerged hollow-fiber membranes have been used for: 1) supplying gas to bioreactors in gas fermentation (Wang et al., 2017); 2) microalgae cultivation (Cheng et al., 2006); 3) nitrification and denitrification (Park et al., 2016); 4) microbial electrosynthesis of chemicals and fuels (Katuri et al., 2016; Alqahtani et al., 2018; Bian et al., 2018; Katuri et al., 2018); and 5) solid-liquid separation to increase the biomass concentration in a chain elongation bioreactor (Kim et al., 2018). To the best of our knowledge, submerged hollow-fiber membranes have not been utilized for pertraction. Using submerged hollow-fiber membranes for MCCA extraction may offer several advantages compared to external hollow-fiber membranes, such as: 1) reduction in the footprint and capital cost; and 2) circumvention of the heating requirement for the recycle broth to and from the external membrane, and thus reducing energy consumption for heating. However, it may be challenging to maintain an equal pressure on both sides of the membrane (i.e., inner side exposed to the hydrophobic solvent and outer side exposed to the fermentation broth in the bioreactor), and to prevent membrane biofouling due to natural attachment and growth of microbes on the surface of the membrane.

Thus, the objective of this study was to develop a chain-elongation bioreactor system with submerged hollow-fiber membranes for pertraction, which can produce and extract MCCAs steadily at a high mass transfer rate for a long period. To address this objective we, first, designed a submerged hollow-fiber membrane bioreactor and tested the system with a synthetic broth for optimizing the operating parameters and extraction rate under abiotic condition. Second, we included the microbiology, and applied a biogas recirculation and a broth recirculation in the fermentation bioreactor to minimize or prevent membrane biofouling. We investigated the effect of these anti-fouling strategies on the MCCA extraction rate, carbon conversion rate, and microbial community. Third, we added an external hollow-fiber membrane module (i.e., traditional pertraction), while maintaining the internal hollow-fiber membranes (i.e., submerged hollow-fiber membranes) to compare the extraction of MCCAs for both extraction systems from the same fermentation bioreactor.

## Materials and Methods

### Substrate and Inoculum

The synthetic basal medium for the biotic experiments was prepared according to a previous study (Kucek et al., 2016b) with the following exceptions: yeast extract (1 g L^−1^) and sodium bicarbonate (1 g L^−1^). Two different concentration ratios of acetate to ethanol were applied during the nine periods to maintain sufficient ethanol in the influent (Table 1). The pH of the medium was adjusted to 5.50 with 4 M of sodium hydroxide. The synthetic broth was prepared with 3 g L^−1^ of Na_2_SO_4_, 20 mM of acetate, 20 mM of *n*-butyrate, 10 mM of *n*-caproate, and 1 mM of *n*-caproate for the abiotic pertraction experiments. The pH of the synthetic broth was set at 5.5.

**TABLE 1:** Experimental approach and operating conditions for submerged pertraction bioreactor during Periods I to IX.

Periods	Date (Days)	Anti-fouling strategy	Pertraction type	Influent (Ethanol : Acetate)
I	0–50	No	Internal	50:25
II	51–83	Biogas recirculation every 4 h for 1 min at 40 ml min^−1^	Internal	50:25
III	84–132	Biogas recirculation every 6 h for 30 min at 20 ml min^−1^	Internal	50:25
IV	133–149	Biogas recirculation every 6 h for 30 min at 80 ml min^−1^	Internal	50:25
V	150–223	Biogas recirculation every 2 h for 5 min at 150 ml min^−1^	Internal	50:25
VI	224–248	Biogas recirculation every 2 h for 5 min at 150 ml min^−1^	Internal	100:25
VII	249–282	Biogas recirculation every 2 h for 5 min at 150 ml min^−1^	Internal + external	100:25
VIII	283–374	Broth recirculation at flow rate of 300 ml min^−1^	Internal + external	100:25
IX	375–402	Broth recirculation at flow rate of 1,600 ml min^−1^	Internal + external	100:25

The reactor was inoculated with a mixed biomass consisting of mangrove sediments, wastewater sludge, granular sludge, and anaerobic digestion sludge to achieve a high microbial diversity in the mixed inoculum. The mangrove sediment was collected from the King Abdullah Monument area (Thuwal, Saudi Arabia). The wastewater sludge was collected from the wastewater treatment plant at King Abdullah University of Science and Technology. The granular sludge and anaerobic digestion sludge were derived from a full-scale aerobic granular sludge reactor (Ali et al., 2020) and a lab-scale anaerobic digestion reactor (Cheng et al., 2019). Each of the inoculum sources was washed three times in a basal medium, and 100 ml of each inoculum was added to the bioreactor.

### Bioreactor Construction

**FIGURE 1:**
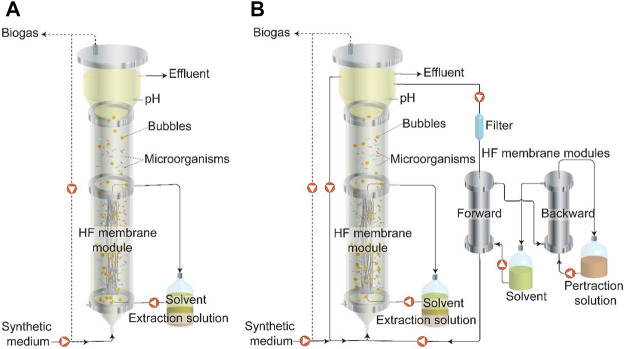
Schematic of two pertraction strategies during Periods I to IX (Table 1): **(A)** Extraction system using only internal hollow-fiber membranes to extract MCCAs during Periods I to VI. Biogas recirculation was applied during Periods II to VI. **(B)** Pertraction system using internal and external hollow-fiber membranes simultaneously to extract MCCAs during Periods VII to IX. Biogas recirculation was applied during Period VII. Broth recirculation was applied during Periods VIII to IX. HF: Hollow Fiber. Dash line represents the gas flow and solid line represents the liquid flow.

The upflow bioreactor consisted of a cylinder with an internal diameter of 5.5 cm and height of 95 cm (Figure 1A and Supplementary Figure 1A), with a working volume of 2.25 L. The temperature of the bioreactor was maintained at 32 ± 1°C using a recirculating water bath (MP-5H, Hinotek, China). The bioreactor broth pH was maintained at 5.5 ± 0.1 by an automatic pH controller (400 pH/ORP, Cole-Parmar, United States) and a dosing pump to add sodium hydroxide solution (2 M). The biogas was collected and recorded by a flow gas meter (TG05, Ritter, Germany). The synthetic medium was continuously fed to the bioreactor from a refrigerated container (4°C), using a peristaltic pump while maintaining an HRT of ∼1 day (Supplementary Figure 2). The effluent continuously exited the bioreactor using an overflow pipe that was fixed near the top of the bioreactor.

### Pertraction System

MCCAs were continuously extracted from the bioreactor with two types of in-line product extraction system with internal and external hollow-fiber membranes as the forward extraction membrane. For the internal hollow-fiber membranes, 4 hollow-fiber membranes (Cleanfil-SMembrane, Kolon Indutries, South Korea) with a length of 44 cm each were assembled as a single bundle, using polyepoxides (Flow-mix, Devcon, United States). One end of the bundle was connected to the bottom port of the bioreactor. The other end of the hollow-fiber bundle was connected to the middle port of the bioreactor. Mineral oil solvent (VWR, United States) with 3% tri-n-octylphosphine oxide (TOPO) (Alfa Aesar, United States) was used as the hydrophobic solvent and it was recycled at an upflow rate of 1 ml min^−1^ (Cerampump, Fluid metering, United States) through the hollow-fiber membranes from a two-phase reservoir in which 200 ml of the hydrophobic solvent and 250–300 ml of the alkaline pertraction solution were phase-separated (Figure 1A). Because of a relatively low expected total rate of extraction, we did not include a backward hollow-fiber membrane module between the solvent and extraction solution. The alkaline extraction solution was initially buffered with 0.2 M boric acid and was maintained at a pH of 9–11 with manual addition of 2 M sodium hydroxide solution every 4–7 days.

For the external hollow-fiber membrane, a forward and a backward membrane module with a contact area of 0.75 m^2^ (MD063CP2N, Microdyn, Germany) were applied as part of a pertraction system, which is similar to those used in a previous study (Figure 1B) (Xu et al., 2018). The bioreactor broth was continuously circulated through the exterior space of the forward membrane module at a flow rate of 50 ml min^−1^. A 5 µm pore size filter (GS-6sed/5, Pentek, United States) was placed before the forward membrane module to prevent membrane module clogging and was replaced every month. The hydrophobic solvent was circulated at a flow rate of 30 ml min^−1^ through the interior of the forward and backward hollow-fiber membrane modules. Due to a higher membrane contact area than the internal hollow-fiber membrane, and therefore a higher overall extraction rate, we used a backward hollow-fiber membrane between the solvent and pertraction solution. An alkaline pertraction solution (2.5 L) from a well-mixed reservoir was circulated at a flow rate of 40 ml min^−1^ through the exterior of the backward hollow-fiber membrane module. This alkaline pertraction solution was similar to the one used for the internal hollow-fiber membrane pertraction.

### Experimental Periods

To reduce membrane fouling, two operating strategies were adopted (Table 1): biogas recirculation (Periods II to VII) and broth recirculation (Periods VIII to IX). During Period I (start-up phase), the bioreactor was operated for 50 days without any anti-fouling treatment. During Periods II to VII, successive cycles of biogas recirculation were varied, including the settling time, flow rate, and time of recirculation (Table 1). During Periods VIII and IX, the bioreactor broth was recirculated to reduce membrane fouling at an upflow rate of 300 ml min^−1^ (7.6 m h^−1^) or 1,600 ml min^−1^ (40.5 m h^−1^), using a gear pump (MG200-400, Fluid-o-Tech, Italy) and a variable frequency drive (JNEV-201-H1FN4S, Teco-Westinghouse, United States). To compare the extraction efficiency between the internal and external hollow-fiber membranes, the two types of product extraction systems were operated in parallel during Period VII to IX (Figure 1B; Table 1). Each period was operated for at least 20 × HRT, and the average HRT and organic loading rate (OLR) were reported (Supplementary Figure 2).

During the abiotic internal hollow-fiber membrane experiments, a carboxylate synthetic solution was continuously fed to the abiotic internal hollow-fiber membrane reactor. We investigated the mass transfer coefficient, and the effects of the solvent-alkaline solution interfacial area on mass transfer rate. Two interfacial areas of 62.4 and 181.8 cm^2^ were conducted during Stage A and Stage B, respectively. An H-type glass container and a cell culture flask were used for the abiotic pertraction experiment with interfacial areas of 62.4 and 181.8 cm^2^, respectively. For the biotic pertraction experiment, only the H-type glass container was used because increasing the interfacial area did not affect the mass transfer rate.

### Microbial Community Analysis

Biomass samples for Illumina 16S rRNA gene sequencing analysis were collected from the bioreactor mixed broth during Periods I to IX (Days 25, 68, 110, 137, 211, 247, 277, 325, and 380) with one sample per period. Biomass samples were collected from a sampling port that was located one-third from the top of the bioreactor. The bioreactor mixed broth was collected in 2 ml centrifuge tubes and centrifuged at 10,000 × g for 10 min to obtain a pellet. The obtained biomass pellets were stored at −80°C until further analysis.

Genomic DNA extraction, DNA amplification, and sequencing were performed according to the protocol in a previous study (Alqahtani et al., 2021). Operational taxonomic unit (OTU) abundance was estimated at 97 identities using the usearch (v. 7.0.1090-usearch_global) (Bian et al., 2021). Taxonomy was assigned to representative OTUs using the RDP classifier in QIIME (Caporaso et al., 2010). A heatmap was created to represent the top 20 OTU using the ggplot package in R.

### Liquid Sampling*,* Analytical Procedures*,* and Calculations

The bioreactor-broth samples were collected every other day directly from the sampling port. The samples were filtered through a 0.22 µm pore filter prior to the analyses of carboxylic acids and ethanol. The composition of carboxylic acids and ethanol was determined with a gas chromatograph (GC) (6890A Series, Agilent Technologies Inc., United States) as described previously (Usack and Angenent, 2015). The concentrations of methane, carbon dioxide, and hydrogen in the biogas were measured weekly using a GC (module 310C; SRI Instruments, United States) as previously described (Alqahtani et al., 2021). Detailed information on the calculations is provided in the Supporting Information (Supplementary Eqs 1–4).

## Results and Discussion

### Operation of Internal Hollow-Fiber Membranes With Abiotic Synthetic Broth

The objective of this work was to demonstrate the technical feasibility of utilizing submerged (i.e., internal) hollow-fiber membranes in the bioreactor as a proof-of-concept for MCCA extraction. The use of an external hollow-fiber membrane module for pertraction has been previously applied where a hydrostatic pressure of 0.5–3 psi has been applied to both the broth side and extraction side across the membranes by adjusting a valve to prevent organic solvent from transferring into the fermentation broth or extraction solution, respectively (Xu et al., 2015; Kucek et al., 2016a; Kucek et al., 2016b; Kucek et al., 2016c; Xu et al., 2018). However, it is difficult to apply a hydrostatic pressure within the atmospheric bioreactor in the case of submerged hollow-fiber membranes in the bioreactor. To circumvent this problem, the hollow-fiber membranes were placed at the middle-to-bottom of the bioreactor (hydrostatic pressure: 0.7–1.3 psi, Figure 1). Steady operation was successfully achieved using abiotic synthetic broth with the exception of organic solvent leaking during the first 2 days.

Several factors affected the steady-state operation of the product extraction system and the extraction rate of MCCAs, including forward and backward contactor area, the flow rate of organic solvent and alkaline solution, type of organic solvent, etc. (Kucek et al., 2016b; Saboe et al., 2018). MCCA extraction included two steps: 1) MCCAs transferring from the broth to the organic solvent (forward); and 2) MCCAs transferring from the organic solvent to the extraction solution (backward). For backward MCCA extraction, an alkaline extraction solution is used to supply a pH (and thus a carboxylic acid concentration) gradient as a driving force for extraction (Xu et al., 2021). In the current study, the two phases of alkaline extraction solution and organic solvent contacted each other directly without any membrane separator for backward extraction (Figure 1A). To determine whether the step of backward MCCA extraction limits the mass transfer in the extraction system, we applied two different contactor areas of 62.4 and 181.8 cm^2^ during Stage A and B, respectively. During Stage A, the stable mass transfer of acetate, *n*-butyrate, *n*-caproate, and *n*-caprylate were obtained at an extraction rate of 4.6, 20.8, 82.2, and 50.4 mmol C m^−2^ d^−1^, respectively (Supplementary Figure 3). Increasing the contactor area to 181.8 cm^2^ during Stage B did not affect the carboxylate extraction rates (Supplementary Figure 3), indicating that the contactor area of 62.4 cm^2^ for alkaline extraction solution and organic solvent was large enough for this extraction system. Indeed, in a previous study it has been reported that the process of backward extraction was not the limiting step when using the same contactor area of forward and backward extraction (Kucek et al., 2016b).

Utilizing an apolar solvent can selectively extract longer carbon chain carboxylic acids and avoids the removal of SCCAs, which are used as an electron acceptor and carbon source for chain elongation. In this study, we used a mixture of mineral oil (apolar) and 3% TOPO (polar) as organic solvent, which has been previously applied to extract MCCAs from fermentation reactor (Ge et al., 2015; Xu et al., 2015; Kucek et al., 2016b; Urban et al., 2017; Xu et al., 2018; Carvajal-Arroyo et al., 2020; Xu et al., 2021). The mineral oil has a low toxicity and a food-grade oil can be used in the food industry (Saboe et al., 2018). We observed that mineral oil mixed in the fermentation bioreactor for a short period did not affect the biological conversion processes (Xu et al., 2015). The high viscous mineral oil can lower the risk of organic solvent transferring into the bioreactor. Although higher partition coefficients of the solvents for MCCAs, such as propiophenone and 2-undecanone, were previously observed (Saboe et al., 2018), it probably has a negative impact on conversion of substrate to MCCAs in the fermentation bioreactor once these solvents dissolved in the bioreactor. The addition of TOPO as an extractant can achieve a high equilibrium constant and increases the solvent affinity for carboxylic acid due to the polarity of its P-O bond (Angenent et al., 2018; Saboe et al., 2018; Carvajal-Arroyo et al., 2020).

### The Effect of Anti-Membrane Fouling Strategies on Medium-Chain Carboxylic Acid Production Rate and Extraction Rate by Internal Hollow-Fiber Membranes

**FIGURE 2:**
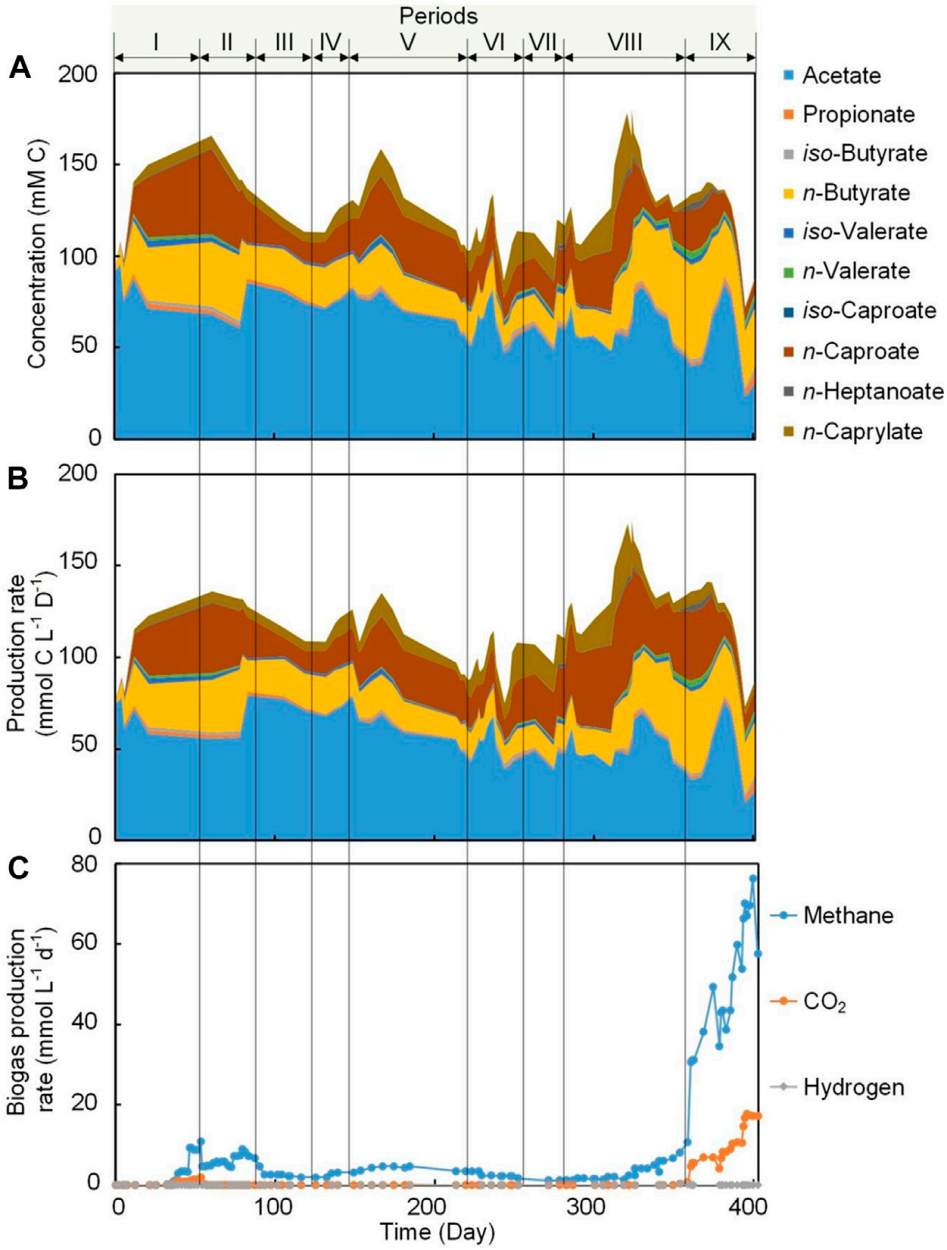
Carboxylic acids concentration in the bioreactor broth and biogas production during Periods I to IX. **(A)** Stacked area chart for broth concentration of carboxylic acids (cumulative). **(B)** Stacked area chart for a production rate of carboxylic acids including effluent, internal extraction and external extraction (cumulative). **(C)** Line chart for biogas production rate (non-cumulative).

Two antifouling strategies were evaluated in this work, which were periodic biogas recirculation (Period II to VII) and broth recirculation (Period VIII to IX) (Table 1). During Period I, with no application of any of the antifouling strategies, we achieved an extraction rate of 100.2 ± 52.2 for *n*-caproate and 77.6 ± 7.2 mmol C m^−2^ d^−1^ for *n*-caprylate by the internal membranes. Introducing periodic biogas recirculation during Period II, resulted in an unexpected decrease in MCCA extraction rates to 66.6 ± 8.4 and 50.4 ± 28.0 mmol C m^−2^ d^−1^ for *n*-caproate and *n*-caprylate, respectively. Several factors might have been responsible for this decrease in MCCA extraction rate as explained below. The volatile solids (VS) decreased from 5.2 ± 0.2 to 3.9 ± 0.01 g L^−1^ (Supplementary Figure 4) due to biomass washout when biogas recirculation was applied (Sankaran et al., 2018), and this in turn might have resulted in the decrease of the concentration of *n*-caproate (from 31.4 ± 13.0 to 18.2 ± 8.9 mM C) and *n*- caprylate (from 6.2 ± 2.1 to 5.2 ± 0.4 mM C) (Figure 2A). Decreases in MCCA extraction rates had been previously observed when the concentration of uncharged MCCAs decreased in the fermentation broth (Kucek et al., 2016a; Carvajal-Arroyo et al., 2020). The VS decrease also resulted in the decrease in MCCA production from 35.9 ± 13.4 mmol C L^−1^ d^−1^ during Period I to 23.4 ± 9.1 mmol C L^−1^ d^−1^ during Period II (Supplementary Table 1). Periodic biogas recirculation (Table 1) was continued during Period III to Period V and the highest MCCA extraction rate of 274.8 ± 78.0 mmol C m^−2^ d^−1^ was obtained during Period IV. During Period IV, the application of biogas recirculation every 6 h for 30 min at a flow rate of 80 min min^−1^ and an ethanol:acetate ratio of 50:25 (mol:mol) was considered the optimum condition for MCCA extraction in this system. Biogas recirculation was applied with the submerged membrane system not only to scour the outer membrane surface and induce a shear force at the membrane surface to remove the accumulated foulants (Fulton et al., 2011; Vermaas et al., 2014), but also to induce a turbulent flow that can increase mass transfer rates (Laptev et al., 2015). Mathematical modelling analysis should be used in future studies to describe and understand the effect of the performing conditions on the process of mass transfer.

To increase the MCCA production rate, the influent concentration of ethanol was doubled to 100 mM during Period VI (Table 1). Enough ethanol (75.2 ± 8.9 mM C) was present in the broth as an electron donor and carbon source to sustain a promising chain elongation rate (Supplementary Figure 5). The average MCCA extraction rate of 655.2 ± 86.4 mmol C m^−2^ d^−1^ (372.0 ± 36.0 mmol C C6 m^−2^ d^−1^ and 283.2 ± 50.4 mmol C C8 m^−2^ d^−1^) obtained here were higher than the rates reported in previous chain elongation studies using external membrane pertraction (Supplementary Table 2), even though the total removal rate per volume of bioreactor was considerably lower. Carvajal-Arroyo et al. (2020) reported a MCCA mass flux of 11.1 g m^−2^ d^−1^ (the aggregated reported number of MCCAs does not allow comparison with a molar unit), while an average maximum MCCA mass flux of 12.2 g m^−2^ d^−1^ was achieved in the present study, which was slightly higher. A regular offline cleaning (once every 3–5 weeks) for the external membrane module by flushing with water to remove the accumulated foulants was performed in previous studies (Xu et al., 2015; Xu et al., 2021), while a continuous high extraction rate was achieved in this work at least for 248 days by regularly recirculating without any offline washing or application of anti-fouling chemical agents. The broth biomass concentration in this work (Supplementary Figure 4) was relatively lower compared to most previous studies (Ge et al., 2015; Xu et al., 2018), and it has been reported that increase in biomass could result in an increase in conversion rate and concentration of products (Xu et al., 2018; Červeňanský et al., 2020), thus, further leading to a high extraction rate (Kucek et al., 2016a; Carvajal-Arroyo et al., 2020). Therefore, increasing the biomass concentration (e.g., adding carriers) would result in further increases in the MCCA extraction rate using internal membrane, albeit probably negatively affecting membrane fouling.

During Period VIII, the broth was recirculated at a flow rate of 300 ml min^−1^ (upflow velocity of 7.6 m h^−1^) to reduce hollow-fiber membrane fouling with an internal and external hollow-fiber membrane system operated in parallel (Table 1). A higher surface-corrected MCCA extraction rate by the internal hollow-fiber membranes (212.2 ± 66.8 mmol C m^−2^ d^−1^) was observed during Period VIII (broth recirculation with two types of pertraction operated in parallel) compared to 166.4 ± 92 mmol C m^−2^ d^−1^ during Period VII (biogas recirculation with two types of pertraction operated in parallel) (Table 2). The results indicated that the extraction rate during operation with broth recirculation was higher than operation with biogas recirculation when internal and external membranes extraction were operated in parallel. Even though a similar fouling strategy (biogas recirculation rate and frequency) was applied during Period VI and VII and a similar ethanol:acetate ratio, the extraction rate was significantly higher during Period VI (655.2 ± 86.4 mmol C m^−2^ d^−1^) than Period VII (166.4 ± 92 mmol C m^−2^ d^−1^), possibly because only internal hollow-fiber membrane extraction (1.5% membrane area of external membrane area) was applied during Period VI compared to internal and external extraction during Period VII. Increasing the broth recirculation rate to 1,600 ml min^−1^ (40.5 m h^−1^) during Period IX resulted in a considerable decrease in the MCCA extraction rate to 110.0 ± 25.0 mmol C m^−2^ d^−1^ by internal hollow-fiber membrane compared to 212.2 ± 66.8 mmol C m^−2^ d^−1^ during Period VIII (broth recirculation rate of 300 ml min^−1^). Under a higher broth upflow velocity, the concentrations of *n*-caproate and *n*-caprylate in the broth decreased to 7.6 and 3.9 mM C, respectively (Figure 2A). The decrease in extraction rate was likely observed due to the low MCCA concentration in the broth, because more substrates were converted to methane than MCCA during this period (Figure 2C).

**TABLE 2:** Carboxylates extraction rates with two pertraction strategies during the nine periods.

Periods	Acetate extraction rate (mmol C m^−2^ d^−1^)	*n*-Butyrate extraction rate (mmol C m^−2^ d^−1^)	*n*-Caproate extraction rate (mmol C m^−2^ d^−1^)	*n*-Caprylate extraction rate (mmol C m^−2^ d^−1^)	MCCAs extraction rate (mmol C m^−2^ d^−1^)
Internal extraction					
I	19.4 ± 2.4	44.8 ± 20.8	100.2 ± 52.2	77.6 ± 7.2	177.8 ± 59.4
II	5.2 ± 2.2	5.2 ± 1.6	66.6 ± 8.4	50.4 ± 28.0	117.0 ± 36.4
III	14.6 ± 6.2	24.0 ± 5.6	121.8 ± 46.2	53.6 ± 2.4	175.4 ± 48.6
IV	12.2 ± 0.3	35.2 ± 4.4	123.6 ± 44.4	151.2 ± 33.6	274.8 ± 78.0
V	3.2 ± 1.0	6.4 ± 2.4	88.8 ± 13.2	48.8 ± 11.2	137.6 ± 24.4
VI	19.2 ± 11.8	57.2 ± 30.4	372.0 ± 36.0	283.2 ± 50.4	655.2 ± 86.4
VII	11.8 ± 4.0	23.6 ± 7.2	84.0 ± 48.0	82.4 ± 44.0	166.4 ± 92.0
VIII	14.6 ± 2.8	29.2 ± 3.2	112.2 ± 37.2	100.0 ± 29.6	212.2 ± 66.8
IX	6.0 ± 3.0	15.2 ± 12.0	30.0 ± 11.4	80.0 ± 13.6	110.0 ± 25.0
External pertraction					
VII	—	2.2 ± 0.6	34.2 ± 5.4	16.0 ± 1.6	50.2 ± 7.0
VIII	0.4 ± 0.04	4.4 ± 0.4	56.4 ± 6.6	8.0 ± 0.8	64.4 ± 7.4
IX	0.3 ± 0.06	1.3 ± 0.3	12.0 ± 3.6	9.6 ± 3.2	21.6 ± 6.8

### Comparison of Internal and External Pertraction on Medium-Chain Carboxylic Acid Extraction Rates and Production Rates

To evaluate the extraction efficiency of internal membranes, we incorporated an external pertraction and operated it in parallel with internal membranes to extract MCCAs from the fermentation reactor during Period VII to IX. During these periods, the extraction rate of *n*-caproate and *n*-caprylate by internal membranes was 2.0- to 2.5-fold and 5.2- to 12.5-fold higher than by external pertraction when corrected to the membrane surface area, but not when corrected to total amount extracted and when corrected to the volume of the broth, respectively (Table 2). The results indicated that the MCCA extraction efficiency as expressed as surface area of membrane only by the internal membranes was much higher than by external pertraction with the same chain elongation bioreactor (Figure 1B). It has been reported that MCCA mass-transfer limitations were at the interface of the fermentation broth and the hydrophobic membrane contactor in the external pertraction system, which was similar to the one used in the present work, and increasing the recycle flow rate of fermentation broth increased MCCA mass transfer (Kucek et al., 2016b). For the current study, a broth recycle flow rate of 3 L h^−1^ (1.6 m h^−1^) was applied in the external pertraction system, which was similar to broth recycle flow rates of 1.5–5.8 L h^−1^ (0.9–3.4 m h^−1^ ) that were used for previous studies (Kucek et al., 2016b; Xu et al., 2018; Carvajal-Arroyo et al., 2020; Xu et al., 2021). For the internal extraction system, we applied both biogas recirculation (Period VII) and broth recirculation (flow rate of 300 ml min^−1^ during Period VIII and 1,600 ml min^−1^ during Period IX) to minimize fouling of the membrane and increase mass transfer. Thus, increased biogas recirculation or broth recirculation flow rate could result in higher MCCA mass transfer rates from the fermentation broth to the hydrophobic solvent. The lower footprint and energy consumption are additional advantages of using an internal extraction system compared to the external pertraction system.

The ratio of membrane area-to-reactor volume for the internal membranes was only 0.004 m^2^ L^−1^, which was much lower than the ratio (0.35–2.5 m^2^ L^−1^) for external pertraction that had been reported for previous studies (Kucek et al., 2016b; Xu et al., 2018; Xu et al., 2021). For the current study, the MCCA extraction efficiency using internal membranes was only 1.8–11.6% during all periods. Therefore, the ratio of extraction membrane area-to-reactor volume was increased to 0.33 m^2^ L^−1^ by operating an external membrane module in parallel with the internal membrane module during Periods VII to IX. The addition of external extraction increased the MCCA extraction efficiency to 35.3–40.7% during Periods VII to IX. The MCCA production rate was also increased from 27.5 mmol C L^−1^ d^−1^ during Period VI (biogas recirculation only, no external pertraction) to 46.5 mmol C L^−1^ d^−1^ during Period VII (biogas recirculation only, with external pertraction), and the highest production rate of 52.7 mmol C L^−1^ d^−1^ was obtained during Period VIII (broth recirculation only, with external pertraction) (Figure 2B; Supplementary Table 1). These results indicate that continuous extraction can lead to an increase in MCCA production rate due to reducing the MCCA cell toxicity and end-product feedback inhibition in the fermentation bioreactor.

### The Effect of Anti-Membrane Fouling Strategies on Biomass Concentration and Microbial Community Composition

In the current study, the VS concentration in the fermentation bioreactor decreased from 5.2 ± 0.2 to 3.9 ± 0.01 g L^−1^ (Supplementary Figure 4) when biogas recirculation was applied during Period II. The VS remained stable at 3.6–4.0 g L^−1^ during Period II to VII with different biogas recirculation frequencies, durations, and flow rates. The VS concentration significantly decreased from 3.6 ± 1.1 to 1.5 ± 0.2 g L^−1^ when a broth recirculation rate of 300 ml min^−1^ (Period VIII) was applied. High biomass concentrations in the fermentation bioreactor are commonly considered to achieve high production rates (Carvajal-Arroyo et al., 2019). Such high concentrations of biomass in the chain elongation reactor can be achieved by: 1) using packing material or settlers (Grootscholten et al., 2013; Kucek et al., 2016b; Liu et al., 2017); 2) forming a chain elongation granular sludge (Roghair et al., 2016; Carvajal-Arroyo et al., 2019); and 3) using a membrane to prevent biomass washout (Kim et al., 2018). Therefore, improving reactor design to enhance biomass retention would result in a higher production rates, however, higher biomass concentration might enhance membrane fouling and future studies should evaluate the maximum biomass concentration required to achieve good production rate and MCCA extraction by internal hollow-fiber membranes without elevating membrane fouling.

**FIGURE 3:**
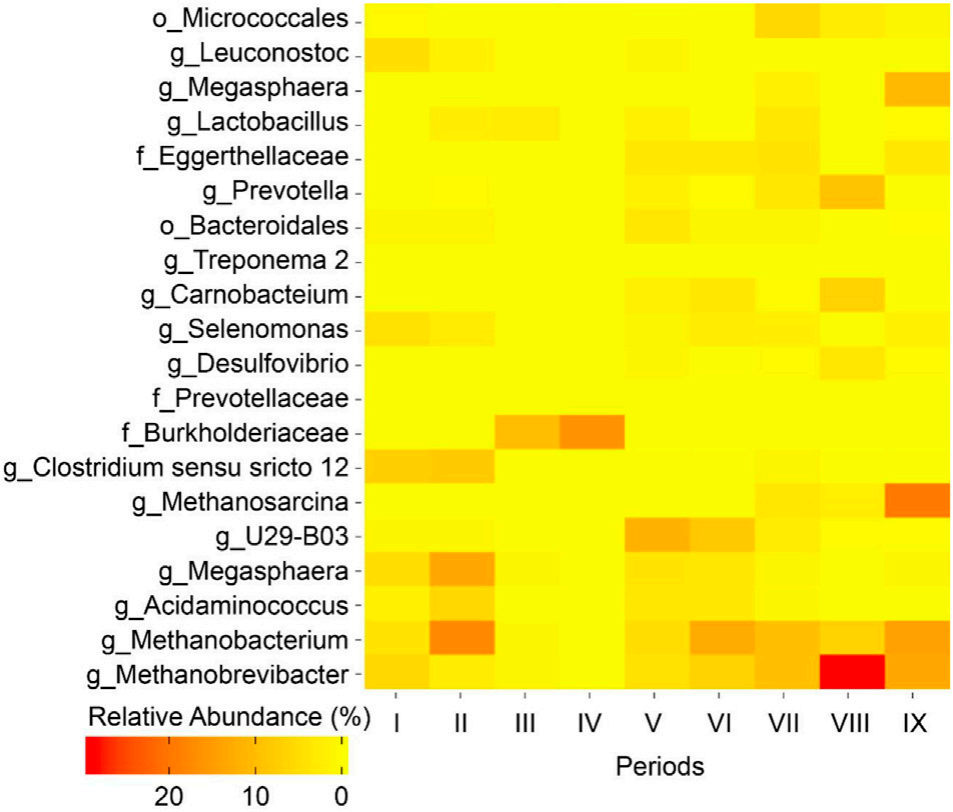
Heatmap of relative OTU abundances of the nine microbiome samples collected during Periods I to IX. The top 20 OTUs with relative abundance ≥1% for one or more of the microbiome samples are listed. The OTUs are classified down to the lowest taxonomic level (o, order; f, family; g, genus) possible.

Methanogens exist in nearly every conceivable anaerobic environment and these microbes can convert organic substrates effectively into methane because it has the lowest free energy content per electron (Zinder, 1993; Angenent et al., 2016). To establish an MCCA production process in open-culture fermentations, one successful option for inhibition of methanogenic activity was maintaining an acidic pH of approximately 5.5 in the fermentation broth (Xu et al., 2015; Kucek et al., 2016c; Xu et al., 2018). In the current study, the pH was maintained at 5.5 and conversion efficiency into methane was low (0.7–5.6% of ethanol and acetate conversion into methane, mol C/mol C; Figure 2C; Supplementary Table 1; Supplementary Eq. 3) during Period I to Period VII (biogas recirculation) despite the fact that members of hydrogenotrophic methanogens belonging to the genus *Methanobacterium* and *Methanobrevibacter* were abundant (accounting for 9.0–19.9% of the total reads) (Figure 3). During Period VIII (broth recirculation at an upflow velocity of 7.6 m h^−1^), members of the genus *Methanobrevibacter* (relative abundance of 28.9%), *Prevotella* (relative abundance of 9.0%), and *Methanobacterium* (relative abundance of 6.7%) were the predominant OTUs detected in the bioreactor (Figure 3). The conversion efficiency to methane increased to 17.3% (mol C/mol C, Supplementary Table 1) during Period VIII. When the broth upflow velocity was increased to 40.5 m h^−1^ (Period IX), the methane production rate significantly increased in the biogas (Figure 2C), and conversion efficiency to methane increased to 30.5% (mol C/mol C, Supplementary Table 1). It should be noted that the highest relative abundance (46.7%) of methanogens (*Methanobrevibacter*, *Methanobacterium*, and *Methanosarcina*) was detected during Period IX. These results indicate that at high upflow velocity the microbial community shifted towards methanogens. Here, we hypothesize that the increase in broth recycling resulted in a loss of hydrogen partial pressure due to hydrogen diffusion out of the recirculation broth. In essence, this changed the bioreactor from a chain elongating system to a methanogenic system, even though acetoclastic methanogens were still not present due to the sufficiently low pH of 5.5. In a chain-elongating bioreactor, methanogens are allowed when the CO_2_ is the limiting factor, maintaining a hydrogen partial pressure that is higher than 10^−2^ kPa. Once the hydrogen partial pressure was reduced to less than 10^−2^ kPa due to diffusion, ethanol was oxidized to acetate, lowering the substrate flux to chain elongation (Spirito et al., 2014). This further dropped the partial pressure of hydrogen further to less than 10^−4^ kPa, because chain elongation produces hydrogen. Next, acetate was oxidized to hydrogen and CO_2_ (Spirito et al., 2014). But both were taken up by the methanogens, while CO_2_ became in excess (Figure 2C). Thus, the system became a methanogenic system because methanogens are superior at scavenging H_2_ when enough CO_2_ is present, making H_2_ the limiting factor. Of course the lack of ethanol and acetate explains why less caproate was produced. Even worse is that the low partial pressure of H_2_ further oxidizes caproate to acetate. It is clear that operators should monitor hydrogen partial pressure during changes in the operating conditions to prevent problems of chain elongation.

## Conclusion

Submerged hollow-fiber membranes (internal) in the fermentation bioreactor were able to achieve high surface-corrected MCCA extraction rates for a long period by biogas recirculation and broth recirculation without any offline washing or anti-fouling chemical agent application to remove foulants. However, higher broth upflow velocity led to low concentration of MCCAs in the fermentation broth because of shift in conversion towards methane production. The results obtained here showed that the surface-corrected extraction rate of MCCAs by internal membranes was much higher than by external pertraction (traditional pertraction) in the same bioreactor. The results in this work showed that the concentration of biomass in this system was relatively low. This study was designed as a proof of concept and further optimizations are required such as: 1) optimization of operation parameters, including flow rate of organic solvent and height of hollow fiber, to prevent organic solvent transferring into fermentation broth when the bioreactor scales up; 2) the use of biocarriers for biomass growth and attachment to increase the density of chain elongation microbes and hence conversion rates; and 3) the increase of surface area for the internal membranes. The use of biocarriers may also help in reducing membrane biofouling.

## Data Availability

The datasets presented in this study can be found in online repositories. The names of the repository/repositories and accession number(s) can be found below: https://www.ncbi.nlm.nih.gov/, PRJNA701879.
