# Supplementary material for: Long-Term Continuous Extraction of Medium-Chain Carboxylates by Pertraction With Submerged Hollow-Fiber Membranes
Source: Front Bioeng Biotechnol. 2021 Aug 13;9:726946. doi: 10.3389/fbioe.2021.726946 (PMC8415110; doi:10.3389/fbioe.2021.726946)
Supplement: Supplementary file 1 [file DataSheet1.PDF]

Supplementary Material

1 Supplementary Equations

Product transfer rate (mmol m^{-2} d^{-1}):

$$\frac{m}{S} \quad \text{(Eq. S1)}$$

Where:

m= slope of the increasing specific carboxylate in the pertraction solution against time, mmol d^{-1}

S = area of hollow fiber membrane, m^2

Volumetric production rate (mmol C L^{-1} d^{-1}):

$$\left[\frac{C_{e,n}V}{HRT} + m_i + m_e\right]\frac{M}{V} \quad \text{(Eq. S2)}$$

Where:

$C_{e,n}$= concentration of carboxylic acid in the effluent on day n, mM

V = volume of the reactor, L

HRT = hydraulic retention time on day n, d

m_i = slope of the increasing specific carboxylate in the pertraction solution using internal hollow fiber against time, mmol d^{-1}

m_e = slope of the increasing specific carboxylate in the pertraction solution using external hollow fiber against time, mmol d^{-1}

M = conversion factor from mmol to mmol C; for example, acetic acid was 2

Conversion efficiency into methane (%, mM C/mM C)

$$\frac{P_m}{L_a + L_e} \quad \text{(Eq. S3)}$$

Where:

P_m= methane production rate, mM C d^{-1}

L_a= acetate loading rate, mM C d^{-1}

L_e= ethanol loading rate, mM C d^{-1}

Carboxylates extraction rates by hollow fiber membrane (mmol m^{-2} d^{-1})

$$\frac{C_e}{M} \qquad \text{(Eq. S4)}$$

Where:

C_e= specific carboxylic acid extraction rate in the extraction solution, mmol d^{-1}

M = area of hollow fiber membrane, m^{-2}

2 Supplementary Figures and Tables

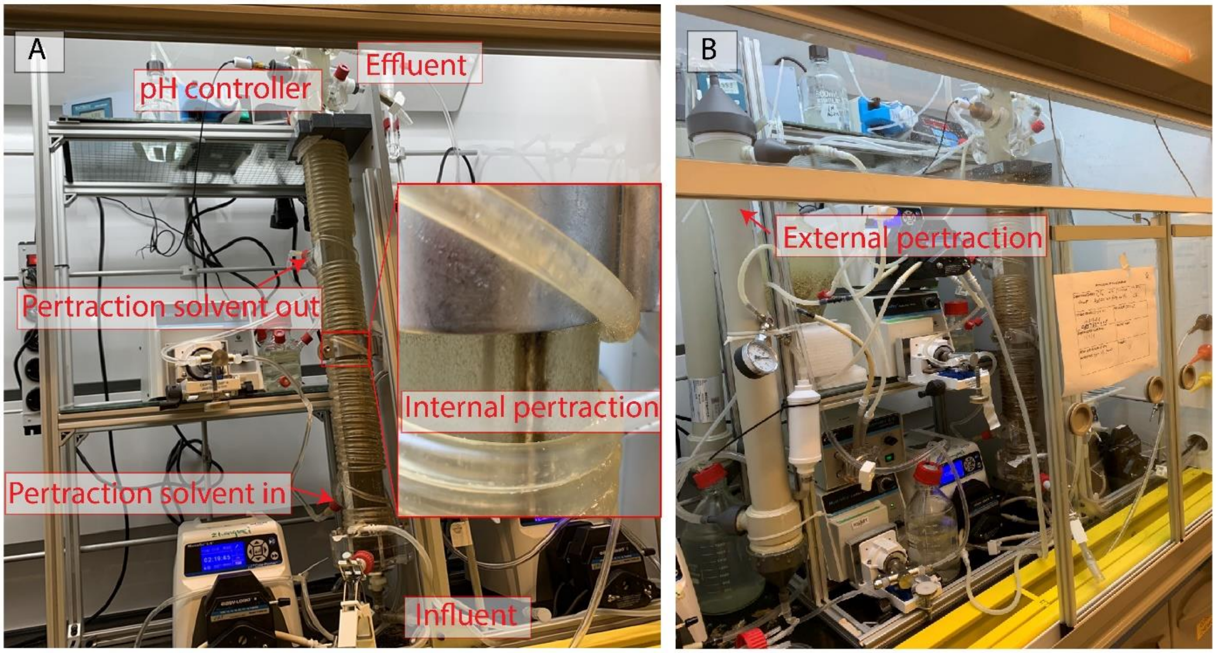

Fig. S1. Photos of the bioreactor and the pertraction systems. (A) The bioreactor combined with pertraction system using internal hollow fiber. (B) The bioreactor combined with pertraction system using internal and external hollow fibers.

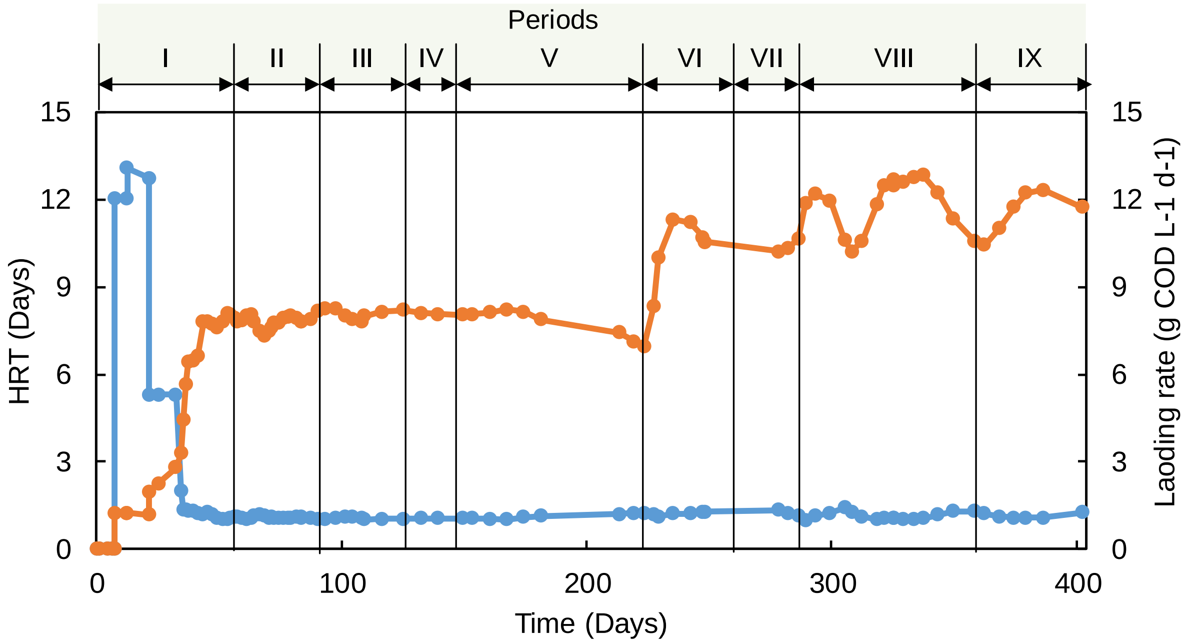

Fig. S2. Hydraulic Retention Time (HRT) and loading rate during Periods I to IX. The blue line represents the HRT and the orange line represents the loading rate.

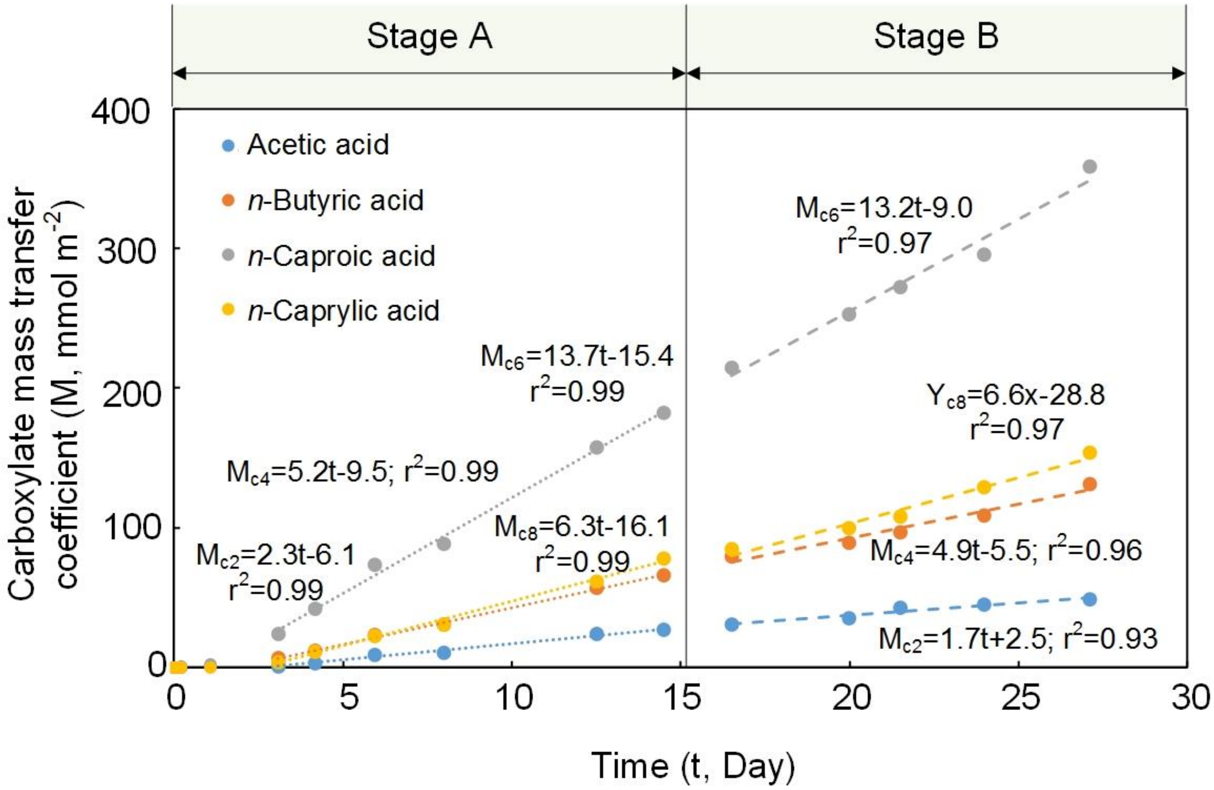

Fig. S3. Carboxylate mass transfer coefficient with abiotic synthetic broth during Stage A and B. C2: acetic acid; C4: *n*-butyric acid; C6: *n*-caproic acid; C8: *n*-caprylic acid.

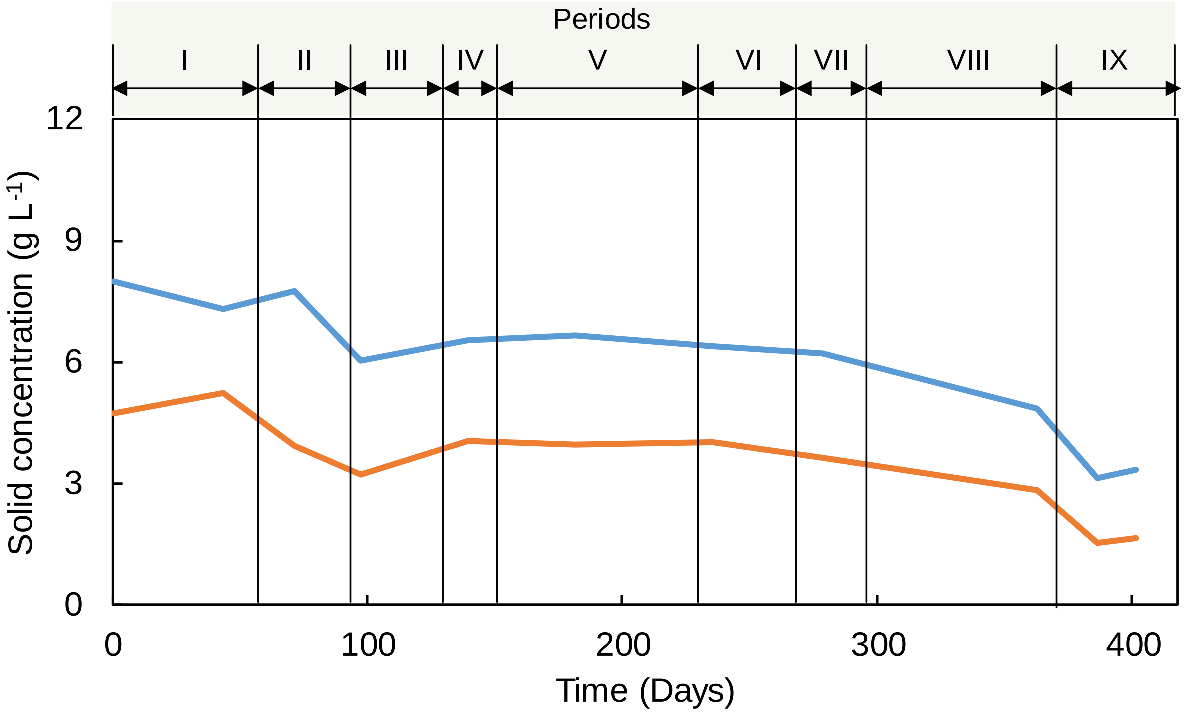

Fig. S4. Solid concentrations during Periods I to IX. The blue line represents the total solid concentration in the effluent. The orange line represents the volatile solid concentration in the effluent.

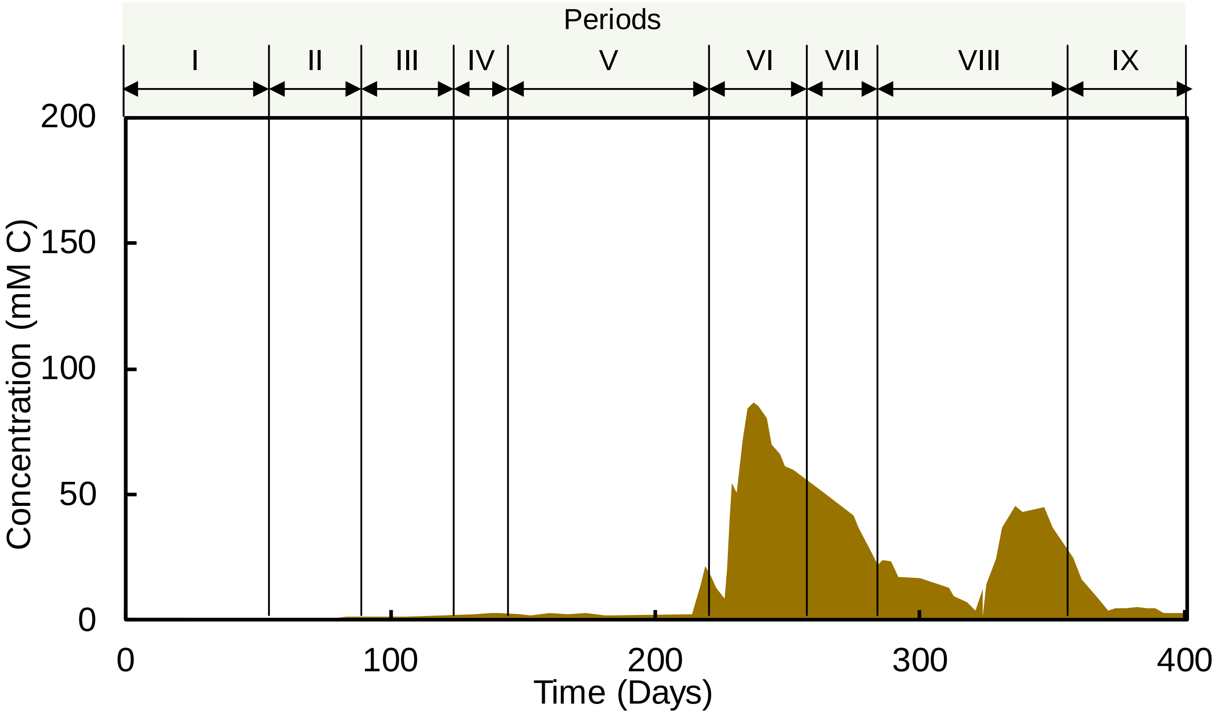

Fig. S5. Ethanol concentration in the effluent during Periods I to IX.

Table S1. Bioreactor production rate and conversion efficiency during the Periods I to IX with internal and external hollow fiber membrane.

	Period I	Period II	Period III	Period IV	Period V	Period VI	Period VII	Period VIII	Period IX
**Volumetric EthOH[1] loading rate (mmol C L^{-1} d^{-1})**	81.3 ± 7.3	92.6 ± 1.7	95.2 ± 1.8	95.2 ± 0.9	84.7 ± 4.3	165.3 ± 8.2	157.5 ± 6.2	166.7 ±15.3	178.6 ± 12.7
**Volumetric Ac[1] loading rate (mmol C L^{-1} d^{-1})**	40.7 ± 3.6	46.3 ± 0.9	47.6 ± 0.5	42.4 ± 2.2	41.3 ± 2.0	39.4 ± 1.6	41.7 ± 3.8	41.7 ± 3.8	44.6 ± 3.2
**EthOH in effluent (mmol C L^{-1} d^{-1})**	0.7 ± 0.2	0.7 ± 0.4	1.7 ± 0.5	2.7 ± 0.3	13.9 ± 3.3	62.1 ± 7.4	34.1 ± 10.1	23.3 ± 13.4	3.5 ± 0.7
**CH_4 production rate (mmol C L^{-1} d^{-1})**	6.9 ± 3.1	7.8 ± 0.8	2.1 ± 0.2	2.7 ±0.7	3.9 ± 0.6	2.4 ± 0.05	1.5 ± 0.5	36.1 ± 8.0	68.1 ± 6.8
**EthOH+Ac-into-CH4 efficiency (% mmol C)**	5.6	5.6	1.4	1.8	3.1	1.1	0.7	17.3	30.5
**CO_2 production rate (mmol C L^{-1} d^{-1})**	1.0 ± 0.4	0.05 ± 0.006	0.01 ± 0.004	0.01 ± 0.001	0.05 ± 0.009	0.03 ± 0.006	0.01 ± 0.001	5.9 ± 1.0	17.2 ± 0.3
**CA[1] production rate (mmol C L^{-1} d^{-1})**	127.5 ± 24.6	113.3 ± 26.7	116.3 ± 14.9	118.8 ± 11.3	89.4 ± 10.7	84.5 ± 12.8	107.2 ± 13.4	138.8 ± 22.1	90.6 ± 21.6
**MCCA[1] Volumetric production rate (mmol C L^{-1} d^{-1})**	35.9 ± 13.4	23.4 ± 9.1	21.0 ± 7.0	25.7 ± 4.8	28.0 ± 7.1	27.5 ± 6.2	46.5 ± 6.8	52.7 ± 6.3	20.4 ± 9.3

[1] EthOH: ethanol; AC: acetate; CA: carboxylic acid; MCCA: medium chain carboxylic acid

Table S2. Performance parameters and MCCA extraction rates of external membrane model pertraction system from previously published studies which were similar to the one used in the present study.

Reference	Type of the broth	Ratio of membrane area to reactor volume (m^2 L^{-1})	Broth recycle flow rate (m hr^{-1})	*n*-Caproate extraction rate (mmol m^{-2} d^{-1})	*n*-Caprylate extraction rate (mmol m^{-2} d^{-1})	MCCAs extraction rate (mmol m^{-2} d^{-1})
6	Filtered bioreactor broth	2.5	1.1	5.3	0.7	6.8
5	Filtered bioreactor broth	0.4	1.0	11.4	11.1	22.5
4	Abiotic synthetic broth	11.6	9.5	6.6	--	--
4	Filtered bioreactor broth	2.0	3.4	3.0	27.2	30.2
1	Filtered bioreactor broth	0.14	1.9	57.8	--	11.1 g m^{-2} d^{-1}
3	Filtered bioreactor broth	2.5	1.6	10.5	--	10.5
2	Filtered bioreactor broth	1.6	--	17.7	--	17.7

References

1. Carvajal-Arroyo, J.M., Andersen, S.J., Ganigué, R., Rozendal, R.A., Angenent, L.T. and Rabaey, K. (2020) Production and extraction of medium chain carboxylic acids at a semi-pilot scale. Chemical Engineering Journal, 127886.
2. Ge, S., Usack, J.G., Spirito, C.M. and Angenent, L.T. (2015) Long-Term n-Caproic Acid Production from Yeast-Fermentation Beer in an Anaerobic Bioreactor with Continuous Product Extraction. Environmental science & technology 49(13), 8012-8021.
3. Kucek, L.A., Nguyen, M. and Angenent, L.T. (2016a) Conversion of L-lactate into n-caproate by a continuously fed reactor microbiome. Water Research 93, 163-171.
4. Kucek, L.A., Spirito, C.M. and Angenent, L.T. (2016b) High n-caprylate productivities and specificities from dilute ethanol and acetate: chain elongation with microbiomes to upgrade products from syngas fermentation. Energy & Environmental Science 9(11), 3482-3494.
5. Xu, J., Guzman, J.J.L. and Angenent, L.T. (2021) Direct Medium-Chain Carboxylic Acid Oil Separation from a Bioreactor by an Electrodialysis/Phase Separation Cell. Environmental science & technology 55(1), 634-644.
6. Xu, J., Hao, J., Guzman, J.J.L., Spirito, C.M., Harroff, L.A. and Angenent, L.T. (2018) Temperature-Phased Conversion of Acid Whey Waste Into Medium-Chain Carboxylic Acids via Lactic Acid: No External e-Donor. Joule 2(2), 280-295.
